# A unique approach to demonstrating that apical bud temperature specifically determines leaf initiation rate in the dicot *Cucumis sativus*

**DOI:** 10.1007/s00425-015-2464-4

**Published:** 2016-01-14

**Authors:** Andreas Savvides, Janneke A. Dieleman, Wim van Ieperen, Leo F. M. Marcelis

**Affiliations:** Horticulture and Product Physiology, Wageningen University, PO Box 16, 6700AA Wageningen, The Netherlands; Wageningen UR Greenhouse Horticulture, PO Box 644, 6700AP Wageningen, The Netherlands

**Keywords:** Cucumber (*Cucumis sativus* L.), Leaf formation, Modelling, Shoot apical meristem

## Abstract

**Leaf initiation rate is largely determined by the apical bud temperature even when apical bud temperature largely deviates from the temperature of other plant organs.**

We have long known that the rate of leaf initiation (LIR) is highly sensitive to temperature, but previous studies in dicots have not rigorously demonstrated that apical bud temperature controls LIR independent of other plant organs temperature. Many models assume that apical bud and leaf temperature are the same. In some environments, the temperature of the apical bud, where leaf initiation occurs, may differ by several degrees Celsius from the temperature of other plant organs. In a 28-days study, we maintained temperature differences between the apical bud and the rest of the individual *Cucumis sativus* plants from −7 to +8 °C by enclosing the apical buds in transparent, temperature-controlled, flow-through, spheres. Our results demonstrate that LIR was completely determined by apical bud temperature independent of other plant organs temperature. These results emphasize the need to measure or model apical bud temperatures in dicots to improve the prediction of crop development rates in simulation models.

## Introduction

Leaf initiation takes place on the shoot apical meristem (SAM), a group of undifferentiated cells usually hidden within young folded developing leaves forming the apical bud. Leaf initiation rate (LIR) determines the number of phytomeres (i.e. shoot module comprised of an internode, a leaf and an axillary bud) formed on a plant per unit of time and thereby strongly affects shoot growth and plant architecture. Consequently, LIR is an important plant trait used in a wide range of plant models where developmental rates, plant leaf area and its distribution along the canopy is of great importance (e.g. Marcelis et al. 1998a; Pallas et al. 2011; Vos et al. 2010; Zhu et al. 2014).

Temperature highly influences LIR (Granier et al. 2002; Granier and Tardieu 1998). LIR linearly increases with the averaged diel temperature in a species-specific range defined by a low (base) and a higher (optimum) threshold temperature (Atkinson and Porter 1996). In fast-growing species, LIR shows relatively steep responses to temperature within this range (*Cucumis melo* L., Baker and Reddy 2001; *Helianthus annuus* L., Granier and Tardieu 1998; *C. sativus* L., Marcelis 1993; *Pisum sativum* L., Turc and Lecoeur 1997). Leaf initiation ceases below the base temperature (Porter and Semenov 2005; Sánchez et al. 2014). Above the optimum temperature, LIR decreases (Craufurd et al. 1998) until leaf initiation ceases again above a maximum temperature (Porter and Semenov 2005; Sánchez et al. 2014).

In most indeterminately growing dicotyledonous crop plants, the apical bud is typically positioned on the top of the shoot. Ambient air temperature (*T*_air_; Craufurd and Wheeler 2009) or plant temperature (Faust and Heins 1993), mostly measured on a single leaf per plant, are typically used to quantify plant developmental responses to temperature although it has been suggested before that it would be more accurate to link LIR to apical bud temperature (*T*_bud_; Craufurd and Wheeler 2009; Granier and Tardieu 1998; Jamieson et al. 1995). Even though plant temperatures usually fluctuate depending on the environment (Jones 1992), this does not necessarily imply that the temperature of a plant always follows *T*_air_. It is well known that leaf temperature often deviates from that of the air due to thermal radiation absorption and transpiration (e.g. Hatfield and Burke 1991) but such deviation from air temperature is usually not considered for other plant organs, though Faust and Heins (1998) and Shimizu et al. (2004) developed biophysical models to simulate shoot tip temperatures of ornamental plants in greenhouses. Recently, it was shown that even under moderate growth conditions the temperature of the apical bud of tomato and cucumber plants significantly deviates from *T*_air_ by several degrees Celsius. This deviation was not constant but varied due to the influences of various climate factors on the thermal balance of the apical bud, as well as due to its ability to transpire (Savvides et al. 2013). This gives rise to the question if it would not be necessary to use the *T*_bud_ instead of *T*_air_ to establish correct relationships between temperature and LIR for plants? This might be of specific importance when changes in plant growth and development are considered in relation to changing climates.

Plant temperature is not always uniform. Vertical intra-plant temperature differences, mainly caused by vertical microclimatic differences, were observed in nature (Gibbs and Patten 1970), field crops (Gardner et al. 1981) and in protected cultivation (Kempkes et al. 2000; Li et al. 2014; Qian et al. 2015). In contrast to other microclimate heterogeneities (e.g. light gradients; Pons et al. 2001), effects of temperature heterogeneities on plant development have hardly been studied. The top of the shoot may be subjected to different solar radiation (Gibbs and Patten 1970), wind speeds (Tuzet et al. 1997) and/or terrestrial (sky and soil) thermal radiation (Leuning and Cremer 1988) than the lower part of the shoot. Therefore, *T*_bud_ may considerably deviate from the temperature of other plant organs (*T*_plant_).

In monocotyledonous plants, such as wheat, the apical bud is located in the crown before the developmental stage of jointing (McMaster et al. 2003). Therefore, before jointing, the temperature of the soil was considered a good approximation of bud temperature in wheat and maize plants (McMaster et al. 2003; Stone et al. 1999). Stone et al. (1999) and McMaster et al. (2003) showed that, under normal conditions, leaf appearance rate is better predicted based on soil than air temperature before jointing. However, McMaster et al. (2003) found that when heating the soil (+3 °C) and not the rest of the plant, LIR did not follow soil temperature. This suggests that *T*_bud_ may not be a good predictor for LIR under bud-plant temperature differences. However, to the best of our knowledge, the effects of bud-plant temperature differences on LIR were not investigated so far.

It can be argued that the temperature of other plant organs would influence LIR under bud-plant temperature differences. For instance, it is well known that environmental cues (e.g. temperature, light intensity, ambient CO_2_ concentration) are sensed by the mature plant tissues (e.g. developed leaves) which generate systemic signals that mediate developmental changes in young tissues (Coupe et al. 2006; Gorsuch et al. 2010; Lake et al. 2001). LIR is known to be influenced by low light intensity (Savvides et al. 2014), increased number of sink organs (Marcelis 1993) or leaf removal (Hussey 1963) suggesting that LIR is dependent on carbohydrate availability. The growth and development of sink organs, like the SAM and the newly formed organs comprising the apical bud, are mostly dependent on the import of carbon from mature leaves (Turgeon 1989). Therefore, the availability of sugars in the apical bud, which is primarily determined by the production of photosynthate at plant level and the partitioning mechanisms between different sink organs (Lemoine et al. 2013), may be a limiting factor for LIR (Savvides et al. 2014). Consequently, altering *T*_plant_ could impact LIR, independent of *T*_bud_. As a result, LIR may not follow *T*_bud_ when the latter is altered from *T*_plant_. The aim of the present study was to investigate how sensitive LIR is for variation of *T*_bud_ and whether LIR is modulated by *T*_bud_ only, or it is also influenced by *T*_plant_ in a crop plant of indeterminate growth. For this, we developed a transparent enclosure around the apical bud with a heating/cooling system in which *T*_bud_, vapour pressure deficit (VPD) and air velocity could be controlled while *T*_plant_ was kept at another level by climate room control (*T*_air_ and VPD_air_). Dicot *C. sativus* L. (cucumber) plants were used as they are fast-growing plants of indeterminate growth.

## Materials and methods

### Plant material and growth conditions

Cucumber (*C. sativus* cv. Venice RZ) plants were grown in a climate room at 22 °C *T*_air_, 70 % relative humidity (RH; VPD = 0.8 kPa) and ~380 μmol mol^−1^ [CO_2_] on rockwool slabs and watered with nutrient solution (EC = 2 dS m^−1^, pH = 5.0–5.5). The plants were illuminated by SON-T lamps (MASTER GreenPower CGT 400 W E40 1SL; Royal Philips Electronics N.V., Amsterdam, The Netherlands) at a photosynthetic photon flux density (PPFD) of 200 μmol m^−2^ s^−1^ during 16 h photoperiods (11.52 mol m^−2^ day^−1^ daily light integral). Two lamps were installed per m^2^ to achieve homogeneous distribution of light intensity. The lamps were isolated from the climate cell by a glass ceiling which enabled the separate convective cooling of the lamps. An energy screen (OLS60; *AB* Ludvig Svensson, Kinna, Sweden) was added below the glass ceiling to reduce the thermal radiation emission by the lamps and maintain the homogeneous distribution of light intensity in the climate room. After the 7th leaf had unfolded (~28 days after plant emergence) and the apical buds appeared as distinct structures on the top of the plant canopy plants were subjected to various bud–plant temperature differences (*T*_bud_ − *T*_plant_).

### Temperature treatments

Plants were subjected to 9 different combinations of *T*_bud_/*T*_plant_ in the range of 18–26 °C (18/18, 22/18, 26/18, 18/22, 22/22, 26/22, 18/26, 22/26, 26/26; Table 1). The differences between *T*_bud_ and *T*_plant_ in a plant (Table 1) were achieved by separately controlling *T*_bud_ (VPD_air_ and air velocity around the bud) using a custom-made device which enclosed the apical bud (see system description below) and maintaining *T*_plant_ (the temperature of the other plant organs) by controlling *T*_air_ and VPD_air_ in the climate room in which the plants were growing. The implicit assumption that in these climate rooms *T*_plant_ strongly depends on *T*_air_ has been verified (see plant and bud temperature measurements below). Set-point temperatures were not all exactly realised but the actual temperatures achieved were substantially close to those set (<1 °C max deviation; Table 1). Eight plants were subjected to each *T*_bud_/*T*_plant_ combination for 28 days. During plant development side shoots were removed when at maximal 2 cm length. In all treatments, fruits were only allowed to develop at every 4th internode starting from the 10th internode to avoid uneven fruit set and abortion and thereby to keep the photosynthate allocation balanced.

**Table 1:** Plant temperature (*T*_plant_) and vapour pressure deficit (VPD) in the ambient air prior to the treatments and during the treatments, apical bud (sphere) temperature (*T*_bud_; *n* = 8), VPD in the sphere, apical bud-based thermal time (*n* = 8) and the difference between *T*_bud_ and *T*_plant_ over the treatments (28 days after the stage of the 7th unfolded leaf)

Treatment (*T*_bud_/*T*_plant_)	Before treatments		During treatments							Sphere vs Ambient	
	Ambient		Ambient			Sphere					
	*T*_plant_ (°C)	VPD (kPa)	Set-point *T*_plant_ (^o^C)	*T*_plant_ (°C)	VPD (kPa)	Set-point *T*_bud_ (°C)	*T*_bud_ (°C)	VPD (kPa)	Thermal time (°C days)	Set-point *T*_bud_ − *T*_plant_ (^o^C)	*T*_bud_ − *T*_plant_ (°C)
18/18	22.0	0.81	18	17.7	0.77	18	18.2^cd^	0.89^a^	229^cd^	0	+0.5
22/18						22	22.1^b^	0.82^ab^	339^b^	+4	+4.3
26/18						26	25.9^a^	0.75^ab^	444^a^	+8	+8.3
18/22	22.1	0.82	22	21.4	0.70	18	18.0^d^	0.65^b^	225^d^	−4	−3.6
22/22						22	22.1^b^	0.63^b^	338^b^	0	−0.7
26/22						26	26.1^a^	0.77^ab^	451^a^	+4	+4.7
18/26	22.2	0.80	26	26.2	0.74	18	18.9^c^	0.86^a^	249^c^	−8	−7.3
22/26						22	22.2^b^	0.87^a^	341^b^	−4	−4.0
26/26						26	26.2^a^	0.79^ab^	452^a^	0	0.0

Different letters within a column indicate significant differences (*P* < 0.05)

### Apical bud heating/cooling system

**Fig. 1:**
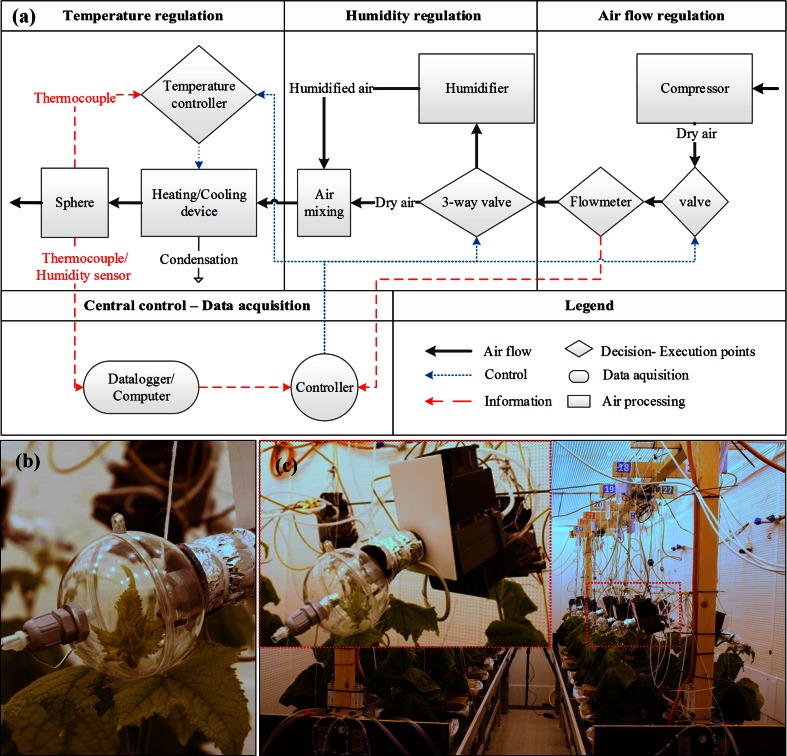
Schematic representation of the heating/cooling system used to alter apical bud temperature in cucumber plants (**a**). Transparent sphere used to isolate apical bud from the ambient environment (**b**). Experimental setup in the climate room and a magnified image of one of the heating/cooling modules attached on a cucumber plant (**c**)

*T*_bud_ in the treatments described above was altered and maintained stable by convective heating/cooling (i.e. changing air temperature locally) using a custom-made heating/cooling (h/c) system (Fig. 1). The VPD and wind speed close to the bud were also controlled to avoid deviations on *T*_bud_ that may occur in cucumber plants (Savvides et al. 2013). After the 7th leaf had unfolded, the apical bud was enclosed within a transparent hollow PVC sphere (Fig. 1b). The sphere was comprised of two hemispheres and allowed ~90 % light transmittance without affecting the light spectrum. To avoid light intensity differences (at apical bud level) between the treatments, the apical buds of all the plants were enclosed in spheres and their *T*_bud_ was controlled by the h/c system. Each sphere was supplied with (humidified) air of certain temperature (18–26 °C). The air was heated/cooled and its temperature was maintained by an h/c device (Fig. 1a, c). The treated air was transported from the h/c device to the sphere via a thermally insulated polyethylene (PE) tube (Fig. 1b). One h/c module (i.e. the combination of a sphere and an h/c device) was used per plant (Fig. 1c). The h/c device was primarily an acrylic chamber via which the compressed air passed through. Through its passage, the air was heated by a heating element or cooled by a Peltier element in the chamber and controlled in an on/off mode by a temperature controller (ET1412 digital thermostat, ENDA, Istanbul, Turkey) located below the h/c chamber (Fig. 1c). Sphere temperature (internal air temperature) was communicated to the temperature controller by a thermocouple (*t*/*c*) inserted into the sphere (Fig. 1a). This allowed the precise regulation and maintenance of the air temperature inside the sphere (Table 1). The air temperature inside the sphere was continuously monitored (every minute) by another t/c connected to a data logger (USB TC-08, Pico Technology, Cambridgeshire, UK) and temperature data were acquired by a computer (Fig. 1a). The 24 h/c devices used (eight per treatment) were electrified by three power supply units (SPS 9400, Maas, Elsdorf–Berrendorf, Germany). The h/c modules should be able to follow the upward movement of the apical buds due to shoot elongation in time. Therefore, the h/c modules were held via wires on a wooden stand on the top of the plants (Fig. 1c) which enabled their individual vertical movement. Young phytomeres with almost unfolded leaves were carefully removed from the sphere by removing one (removable) of the two hemispheres and simultaneously moving the h/c module upwards. H/c module adjustments were taking place twice a day (early in the morning and late in the afternoon).

To maintain similar VPD (~0.8 kPa) in all treatments, the dry compressed air inserted to the h/c system was appropriately humidified prior the entrance in the h/c device. A fraction of the compressed dry air was bypassed through a humidifier. The fraction was controlled manually by a three-way valve (Fig. 1a). The humidifier was a sealed barrel half-filled with de-ionized water (to avoid salt accumulation in the h/c system) via which the air was forced to pass by submerging the cut end of the dry air-bearing PE tube. After humidification, the compressed humidified air was directed via another non-submerged PE tube outside the barrel and mixed in the way to the h/c device using a T-tubing connection with the volume of dry air that bypassed the humidifier. Relative air humidity in the sphere was continuously monitored by a humidity sensor (WS–DLTc, Wireless Value, The Netherlands) and the data were collected by a computer (Fig. 1a). VPD was calculated based on relative humidity and air temperature inside the sphere.

Wind speed in the sphere was maintained at the levels of the ambient wind speed (~0.2 m s^−1^) by controlling the air flow prior the humidification of the compressed dry air (Fig. 1a). Air flow was continuously monitored by an air flow-meter (ENK5FRH, Kutola Instruments, Muurame, Finland) and controlled manually using a valve connected on the PE tubing system before the flow-meter in the direction of flow (Fig. 1a). Ambient and sphere wind speed were measured by a 3d-anemometer (WindMaster™; Gill Instruments LTD, Hampshire, UK) and an air velocity meter (Velocicalc 8347, TSI, MN, USA), respectively, prior the treatments.

### Plant and bud temperature measurements

Temperature measurements with contact K-type thermocouples (t/c’s) on soft meristematic tissue in the apical bud are potentially harmful, especially because the t/c have to be daily repositioned to ensure good contact with the fast-growing tissue. To avoid harming the apical bud and its influence on LIR we did not directly measure *T*_bud_ but used the temperature of the air in the sphere as a proxy for *T*_bud_ assuming negligible differences. This assumption was thoroughly verified in a pilot study prior to the main experiment at three air temperatures inside the sphere enclosing the bud (18, 22 and 26 °C) and further comparable conditions in the climate cell as during the main experiment. *T*_bud_, measured by gently inserting t/c into the centre of the bud, strictly followed the air temperature inside the sphere. Therefore, the air temperature inside the sphere was considered to be similar to *T*_bud_. In a second pilot experiment we tested whether the temperature of the rest of the plant outside the sphere (*T*_plant_) was uniform and comparable to *T*_air_ in the growth chamber. Temperatures of the 9th leaf (mid shoot) and 5th leaf (bottom shoot) were measured by t/c attachment on the abaxial side of the leaf lamina. These leaf temperatures were similar to the ambient air temperature (*T*_air_) in all tests. Therefore, *T*_plant_ was considered to be similar to *T*_air_.

### Leaf initiation rate

LIR was defined as the number of leaves initiated during the temperature treatments divided by the treatment duration of 28 days. Numbers of initiated leaves were obtained by counting (destructive measurements on 8 plants per treatment at both start and end of each treatment). Very young leaves and leaf primordia in the apical bud were quantified by dissecting the apical bud under a stereomicroscope (Wild M7 S, Heerbrugg, Switzerland; 60×–310×). The latest initiated leaf primordium was defined as the latest formed projection that was visible at the side of the meristem (dome). LIR was also calculated per unit thermal time (LIR_dd_; degree (°C) − days) and was based on *T*_bud_. Thermal time (in degree [°C] − days) was estimated based on:

$${\text{Thermal time}} = \mathop \sum \limits_{n = 1}^{k} \left[ {(T_{\text{bud}} )_{n} - T_{\text{base}} } \right]$$

*T*_bud_ is the mean diel temperature of the bud while *T*_base_ is the base temperature at which cessation of the developmental process occurs (Trudgill et al. 2005). *k* is the duration of the treatments in days.

### Statistical analysis

The statistical analysis was performed using SPSS statistics v22.0 for Windows (SPSS IBM, NY, USA). One-way analysis of variance (ANOVA) was used and statistically significant differences on *T*_bud_, VPD in the sphere and LIR_dd_ between treatment means were evaluated with post hoc Tukey’s honestly significant (HSD) multiple comparison tests (*P* < 0.05). The general linear model (PROC GLM) was fitted to the data to test for statistical significance (*P* < 0.05) of the effects of *T*_bud_, *T*_plant_ and their interaction (*T*_bud_ × *T*_plant_) on LIR.

## Results and discussion

**Fig. 2:**
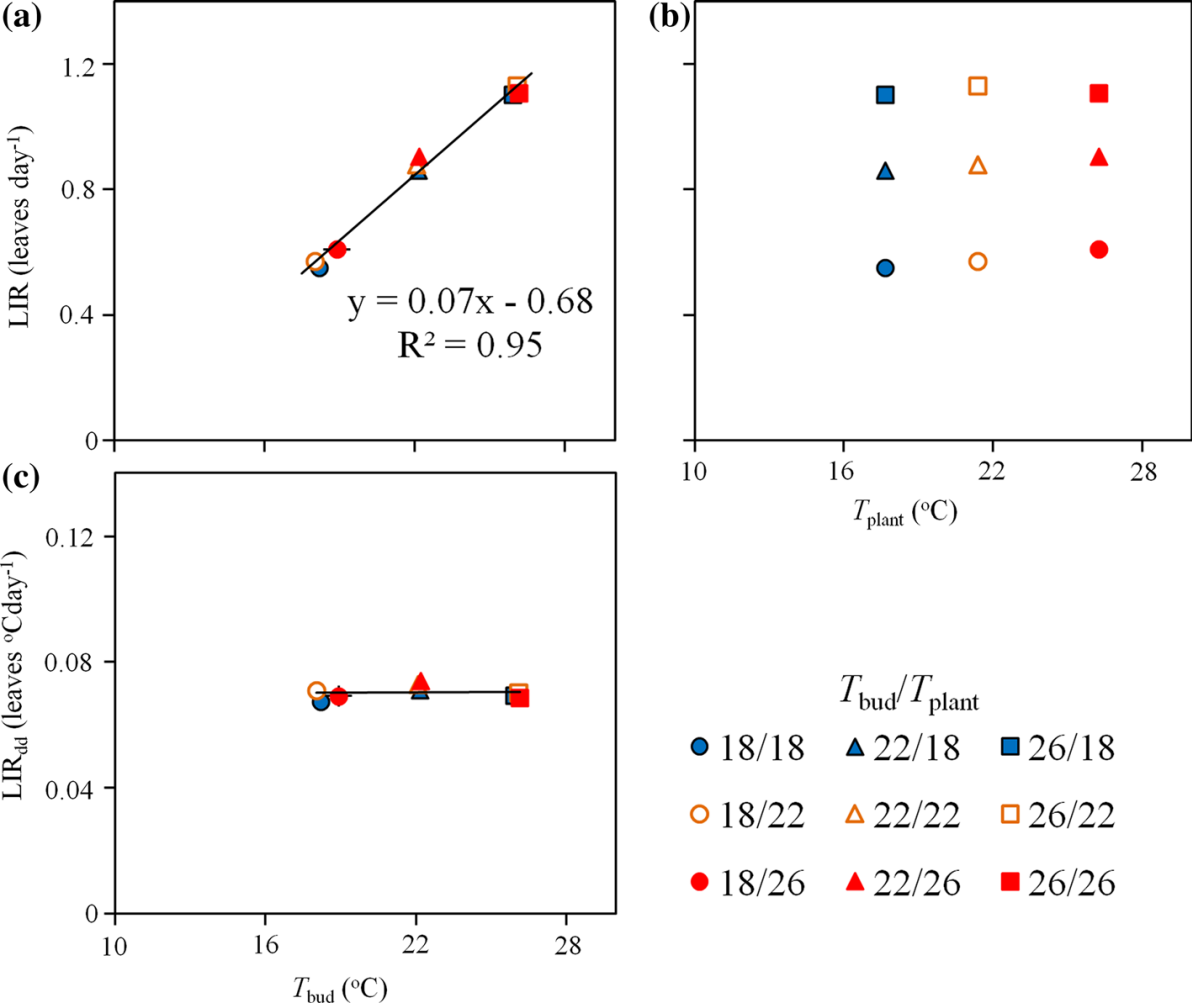
Leaf initiation rate (LIR; *n* = 8) increased linearly (12.1 % per °C) with apical bud temperature (*T*_bud_) in the range of 18–26 °C (**a**) regardless the variations in the temperature of other plant organs (*T*_plant_; **b**). LIR normalized with *T*_bud_-based thermal time (LIR_dd_) did not significantly differ across the treatments (**c**). Values are the means of measurements on 8 plants ± s.e (s.e. smaller than the sample size)

Leaf initiation rate (LIR) in cucumber plants increased with increasing *T*_bud_ in the range of 18–26 °C (*P* < 0.001) and was not affected by *T*_plant_ when varied within the same temperature range (*P* = 0.07; Fig. 2a, b). The sensitivity of LIR for *T*_bud_ was large: it increased linearly with *T*_bud_ at a rate of 12.1 % per °C, while large variation in plant temperature (up to 8 °C; Table 1) did not change LIR when the bud temperature was kept constant, even on the long-term (28 days). The temperatures applied in this study are typical moderate growth temperatures that are not expected to cause any temperature related stress. Present results clearly show that in cucumber small variations in bud temperature already have large consequences for leaf initiation rate. Based on these results it can be concluded that in cucumber only the temperature of the location where leaf initiation actually occurs (the apical bud) is relevant for LIR.

In contrast to our conclusion, McMaster et al. (2003) concluded based on soil-heating experiments in wheat that *T*_bud_ may not be a good predictor for leaf appearance rate (LAR) when bud temperature deviates from plant temperature. The contrast between McMaster et al. (2003) and our study may be due to several reasons. First, McMaster et al. (2003) did not count leaves initiated on the shoot apical meristem but the leaves appeared. LAR is a measure of the speed at which leaves reach a certain early (visible to eye) growing stage while LIR (quantified in this study) is a measure of the speed at which leaves initiate on the shoot apical meristem (SAM; visible using microscopy). Therefore, LAR depends on the speed at which leaves are initiated and expanded at the early stages while LIR depends only on the speed at which leaves are initiated. Leaf initiation and initial leaf expansion are considered as interconnected processes (Savvides et al. 2014). Consequently, it can be speculated that leaf appearance rate can be a good approximation of leaf initiation rate. However, previous studies have shown that both in dicotyledonous (e.g. cucumber; Savvides et al. 2014) and monocotyledonous plants (e.g. wheat; Beemster and Masle 1996) LAR may not sufficiently approximate LIR due to changes in leaf expansion rates during ontogeny. Conclusively, LAR may not follow bud temperature when the latter is deviating from plant temperature but LIR may do so. Second, monocots and dicots may show differences in leaf growth responses to temperature variations (Poiré et al. 2010). Additionally, the influence on LIR may be very different in determinately growing wheat, which stops leaf initiation after jointing (McMaster et al. 2003) and indeterminately growing cucumber, which continuously keeps on forming new leaves. Further research is necessary to explore potential differences between responses of monocots and dicots to bud-plant temperature differences. Third, the different conclusions may be due to differences in the methodologies used. We maintained precisely controlled constant temperatures in the apical buds, carefully checked this and secured that a range of constant bud-plant temperature differences were achieved from −7 to +8 °C. McMaster et al. (2003) quantified soil temperature around the crown of wheat but not the actual bud temperature in wheat plants. They maintained one constant temperature difference between soil and ambient air of +3 °C, while the ambient air temperature changed during the experiment. We (Savvides et al. 2013) have previously shown that apical bud temperature may differ substantially from ambient temperature due to a range of environmental factors which influence the heat balance of apical bud. We also showed that the actually achieved bud temperature is species specific, i.e. differs between species under the same environmental conditions. For example, variations in transpiration rates and related evaporative cooling of the apical bud played an important role in determining the bud temperature (Savvides et al. 2013). It remains uncertain whether bud temperature was proportionally increased with increased soil temperature in the study of McMaster et al. (2003) during the soil-heating experiments. Further research incorporating more than one distinct different plant species and using appropriate methodological approaches may yield useful insights for the discrepancies between present and previous experiments.

After normalizing LIR for thermal time (with thermal time based on *T*_bud_; LIR_dd_), it was not significantly different across treatments (*P* = 0.09; Fig. 2c). Thermal time provides a way for modelling temperature-development relations for poikilotherms (i.e. organism whose temperature fluctuates considerably depending on the environment) such as plants (Granier et al. 2002) and invertebrates (Trudgill et al. 2005). According to this concept temperature-development relations are considered to be linear between a base and an optimum temperature (Trudgill et al. 2005). Therefore, expressing developmental rates in thermal- instead of calendar time would result in normalization for temperature. Backward projection of the linear relation between LIR and *T*_bud_ (Fig. 2a) until zero LIR (see Trudgill et al. 2005) revealed a base temperature (*T*_base_) of 10 °C. In this study the effects of apical bud temperature on LIR were well normalized when LIR was expressed in thermal time (LIR_dd_; Fig. 2c). Consequently, the use of apical bud temperature is the accurate approach for describing temperature effects on LIR.

This study clearly shows that apical bud temperature should be quantified, modelled, predicted and used when studying the rate of leaf initiation (e.g. Chelle 2005; Craufurd and Wheeler 2009; Faust and Heins 1998; Grace 2006; Guilioni et al. 2000; Savvides et al. 2013; Shimizu et al. 2004; Vinocur and Ritchie 2001). We also show the necessity to couple an important developmental process such as leaf initiation, to the temperature actually perceived by the organ in which the process occurs instead of to air- or plant-temperature in growth models. This integration can be progressively achieved first by downscaling microclimate modelling to plant organ instead of canopy (Chelle 2005) and second by integrating to plant level via coupling of microclimate models at organ level using functional structural plant models (Vos et al. 2010). Present findings are also of importance to up-scaling models that are used to simulate plant growth and plant community responses to global climate change. These models combine phenological models with climate change scenarios (Kramer et al. 2000) and may be prone to substantial errors if air temperature is used instead of bud temperature.

This study focuses on temperature responses within the normal-for-growth temperature range (18–26 °C). Studies on LIR-related responses to sub- or supra-optimal temperatures are few (Sánchez et al. 2014 and studies therein) and there are no studies on responses to intra-plant temperature differences within these temperature ranges. Therefore, possible effects of bud-plant temperature differences on plant growth and development within these ranges still necessitate exploration. In addition, a previous study suggested that spikelet sterility in rice because of extreme temperatures can be better predicted based on organ temperature (Julia and Dingkuhn 2013). Consequently, the uncertainty of studies on extreme temperature responses would significantly reduce when organ instead of air temperature is used for modelling temperature responses (Sánchez et al. 2014; Vinocur and Ritchie 2001).

The fact that LIR was influenced by *T*_bud_ and not by the temperature of other plant organs, suggests the absence of a regulating factor outside the apical bud regarding temperature responses. In certain species, which include cucumber and tomato, LIR is also sensitive to factors other than temperature, such as fruit load (*C.sativus* L., Marcelis 1993) and source strength (*Solanum lycopersicum* L., Hussey 1963), suggesting the presence of an external-to-the-apical bud factor regulating LIR, such as carbohydrates. Recent results on tomato and cucumber also show that LIR is only reduced at daily light sums below a threshold of 6.5 mol m^−2^ day^−1^ (Savvides et al. 2014), which is equivalent or less than irradiance levels in winter conditions when growth of most herbaceous plants is arrested or severely reduced. In the present experiment considerably higher light sums were applied and limitations of LIR caused by low irradiance, or related effects on carbohydrate availability, did not play a role.

The control of organ microclimate has been proven important in answering essential questions on plant organ functioning, organ-environment relations and systemic signalling (Coupe et al. 2006; Gorsuch et al. 2010; Lake et al. 2001). In this study, *T*_bud_ was effectively controlled (Table 1) for 28 days using a custom-made apical bud heating/cooling system (Fig. 1). The VPD in the sphere was kept in the range of 0.6–0.9 kPa across treatments (Table 1). Statistically significant differences of VPD in the sphere were observed between some treatments but these differences were neither systematic nor large (Table 1). We here provide a detailed description of a novel methodology that can be constructed by the use of easy-to-find materials and used in future plant organ microclimate research. Even though, the present apparatus as such cannot be used under field conditions (e.g. high radiation will influence sphere’s capacity to cool the apical bud) the methodology behind can be exploited to build field-friendly systems.

## Conclusions

Leaf initiation rates follow apical bud temperature even when the temperature of other plant organs largely deviates from bud temperature. In cucumber plants, LIR shows high sensitivity to apical bud temperature within a moderate temperature range. Consequently, accurate measurements or realistic estimates of *T*_bud_ should be used in experimental and modelling studies in which plant development is a key issue. The present findings add to a better understanding of plant developmental responses to a spatially diverse thermal environment and promote the implementation of this knowledge in future studies.

### *Author contribution statement*

AS, JD, WVI and LM conceived the research. AS designed the research, performed the experiments, analyzed the data and wrote the manuscript. All authors read, commented on- and approved the manuscript.
